# Thrombotic circuit complications during venovenous extracorporeal membrane oxygenation in COVID-19

**DOI:** 10.1007/s11239-020-02217-1

**Published:** 2020-07-11

**Authors:** Xavier Bemtgen, Viviane Zotzmann, Christoph Benk, Jonathan Rilinger, Katrin Steiner, Alexander Asmussen, Christoph Bode, Tobias Wengenmayer, Sven Maier, Dawid L. Staudacher

**Affiliations:** 1 Department of Medicine III (Interdisciplinary Medical Intensive Care), Medical Center—University of Freiburg, Faculty of Medicine, University of Freiburg, Hugstetter Straße 55, 79106 Freiburg, Germany; 2 Department of Cardiology and Angiology I, Heart Center Freiburg University, Faculty of Medicine, University of Freiburg, Freiburg, Germany; 3 Department of Cardiovascular Surgery, Heart Center Freiburg University, Faculty of Medicine, University of Freiburg, Freiburg, Germany; 4 Department of Anaesthesia, Intensive Care Medicine and Pain Medicine, Medical University of Vienna, Vienna, Austria

**Keywords:** V-V ECMO, COVID-19, Thrombotic complications, Pump head thrombosis

## Abstract

The novel coronavirus SARS-CoV-2 and the resulting disease COVID-19 causes pulmonary failure including severe courses requiring venovenous extracorporeal membrane oxygenation (V-V ECMO). Coagulopathy is a known complication of COVID-19 leading to thrombotic events including pulmonary embolism. It is unclear if the coagulopathy also increases thrombotic circuit complications of the ECMO. Aim of the present study therefor was to investigate the rate of V-V ECMO complications in COVID-19. We conducted a retrospective registry study including all patients on V-V ECMO treated at our centre between 01/2018 and 04/2020. COVID-19 cases were compared non- COVID-19 cases. All circuit related complications resulting in partial or complete exchange of the extracorporeal system were registered. In total, 66 patients were analysed of which 11 (16.7%) were SARS-CoV-2 positive. The two groups did not differ in clinical parameters including age (COVID-19 59.4 vs. non-COVID-19 58.1 years), gender (36.4% vs. 40%), BMI (27.8 vs. 24.2) and severity of illness as quantified by the RESP Score (1pt. vs 1pt.). 28 days survival was similar in both groups (72.7% vs. 58.2%). While anticoagulation was similar in both groups (*p* = 0.09), centrifugal pump head thrombosis was more frequent in COVID-19 (9/11 versus 16/55 *p* < 0.01). Neither the time to first exchange (*p* = 0.61) nor blood flow at exchange (*p* = 0.68) did differ in both groups. D-dimer levels prior to the thrombotic events were significantly higher in COVID-19 (mean 15.48 vs 26.59, *p* = 0.01). The SARS-CoV-2 induced infection is associated with higher rates of thrombotic events of the extracorporeal system during V-V ECMO therapy.

## Highlights

- This study compared the rate of thrombotic circuit complications in COVID-19 V-V ECMO patients to historical controls.
- The two groups were comparable.
- Patients with COVID-19 had significantly more thrombotic circuit complications and D-Dimer levels prior to exchange were significantly higher.

## Introduction

The novel severe acute respiratory syndrome coronavirus 2 (SARS-CoV-2) and the related coronavirus disease 2019 (COVID-19) has grown to a worldwide pandemic. Patients infected with SARS-CoV-2 have a great variety of symptoms reaching from asymptomatic course to sever pulmonary impairment [1]. In one epidemiological study reporting 1.099 patients, 15.7% were categorized as “sever” on admission and 37 developed an pulmonary failure comparable to an acute respiratory distress syndrome (ARDS) as proposed by Archer et al.[2, 3]. The mortality seems to be considerable in critically ill patients admitted to the intensive care unit (ICU) as one early study reported a mortality of 61.5% at 28 days [4]. Here, 67% developed an ARDS-like phenotype during the infection with SARS-CoV-2 [4].

Treatment of acute respiratory failure of a COVID-19 infection consists of different pulmonary support strategies: non-invasive ventilation, invasive ventilation, prone position and in selective cases venovenous extracorporeal membrane oxygenation (V-V ECMO) [5]. Initial reports on V-V ECMO utilization during COVID-19 respiratory failure have described a hospital mortality of 50% though newer register data reported by the European Extracorporeal Life Support Organization (EuroELSO) seems to be more encouraging with a reported death rate of 17.1% with a great variety between centers [6, 7].

Although scarcity of resources during a pandemic is a major risk, V-V ECMO is still considered a viable option in selective cases following failure of conventional therapy [5].

Newly emerging data on COVID-19 appears to be associated with a high rate of venous and arterial thromboembolic complications with reported cumulative rates of 21% [8]. To this date, only very limited data is available concerning rate of V-V ECMO complications in this specific patient group. In a large French prospective cohort study, 12 patients with V-V ECMO were reported and a total of three thrombotic occlusions of the centrifugal pump in two patients occurred (8%) [9].

Aim of the present study therefor was to investigate the rate of V-V ECMO complications in COVID-19 infection in comparison to a retrospective control group from 2018 to 2019.

## Methods

Data derives from a registry of all patients on V-V ECMO treated at a medical intensive care unit located at a tertiary university hospital. We offer a 24/7 service for referral of ARDS and ECMO patients. As for local policy, decision to cannulate for V-V ECMO is made after multidisciplinary discussion at the bedside.

### ECMO circuit

Two different pump types were used for ECMO: Stöckert Centrifugal Pump Console (SCPC) (LivaNova, Munich, Germany) and the Cardiohelp-System (Maquet, Rastatt, Germany). For interhospital transportation of patients after ECMO implantation the Lifebox console (LivaNova, Munich, Germany) or the Cardiohelp-System was used. The tubing sets and Oxygenators consists of three sets: HLS 7.0-Set (Priming volume: 273 ml, Surface: 1.8 m^2^) (Maquet, Rastatt, Germany) and two customized tubing sets with a LivaNova EOS ECMO Oxygenator (Priming volume: 150 ml, Surface: 1.2 m^2^) or EuroSets A.L.ONE ECMO Oxygenator (Priming volume: 225 ml, Surface: 1.8 m^2^) (EuroSets, Medolla, Italy). If two oxygenators were used in parallel, Y-connectors were used before and after the oxygenators to split the blood flow. All Sets were fully coated (Maquet: Bioline, LivaNova and EuroSets: Phosphorylcholine). The Priming consists of 700 ml electrolyte solution and 5000 IE unfractionated heparin. In case of heparin-induced thrombocytopenia, no unfractionated heparin was used for priming.

### V-V ECMO management

All ECMO circuits were checked once a day by a perfusionist and three times a day by each a nurse and a physician for visible thrombus formations, lipid deposits, changes of the pump head bearing. Indications for exchange of the whole ECMO-System, except the cannulas, were thrombus formations with the risk of thromboembolic events or system failure. In case of visible thrombus formation at the pump head or running noise an isolated change of the pump head was performed if gas exchange was sufficient and no further thrombus formation in the ECMO circuit was visible. Isolated change of connectors in the tubing set was necessary in case of thrombus formation at a connector with no thrombus at the pump head or oxygenator. All exchanges were carried out jointly by a registered nurse, a perfusionist and an ECMO specialist.

As for this research, only changes in the ECMO circuits requiring an exchange of the pump head or the whole system were considered of clinical importance. Each exchange was judged by a perfusionist and an ECMO physician independently reviewing all available documentation. All non-thrombotic complications requiring exchange were excluded. In case of dissent of the two independent reviewers, the exchange was included in the analysis.

### Anticoagulation

During V-V ECMO therapy, anticoagulation with continuous infusion of unfractionated heparin was administered as defined by standard operating procedure. When there was no other indication for therapeutic anticoagulation, the target range for activated partial thromboplastin time (aPTT) was 40–50 s and adjusted depending on thrombus burden or bleeding events. If there was suspicion or proof of heparin-induced thrombocytopenia or insufficient thrombus control in the circuit with unfractionated heparin, the anticoagulation was switched to argatroban with an aPTT target of > 60 s. As reports of thrombotic complications in COVID-19 emerged, anticoagulation target was increased from aPTT 40–50 s to aPTT 50–70 s.

### Data and ethics

COVID-19 patients were recruited prospectively as was data acquisition. The control group consisted of all V-V ECMO patients treated at our hospital during the years 2018 to 2019 and were recruited retrospectively as was data acquisition. This research was covered by an ethics agreement (ethics committee of the Albert-Ludwigs-University, Freiburg, Germany; file numbers 151/14 and 234–20). Consent was not required as approved by the ethics committee. This study was performed in line with the principles of the 1964 Declaration of Helsinki and its later amendments. Demographic data (age, sex and body dimensions), baseline characteristics related to the V-V ECMO (duration, indication, cannula size), anticoagulation strategy, D-dimers measurements, survival to discharge and presence of complications related to the V-V ECMO device were collected.

### Statistical analysis

For data analysis, SPSS (version 23, IBM Statistics) or Prism (version 8, GraphPad) were employed. For statistical analysis, unpaired *t*-test, Fisher’s-exact/chi-square test, and Log-rank/Gehan Breslow test were used as applicable and a *p* value of < 0.05 was considered statistically significant. All data are presented as absolute number (percent of all patients) or as median with inter quartile range if not stated otherwise.

## Results

A total of 66 V-V ECMO patients were included, eleven patients who were tested positive for SARS-CoV-2 and 55 patients treated at our hospital during the years 2018 and 2019. 28 days survival was 72.7% in the COVID-19 group and 58.2% for the non-COVID-19 group. At the time of conception of the manuscript two COVID-19 patients were still in ongoing V-V ECMO therapy. The median age was 58.4 (46.9–66.2) years at time of V-V ECMO implantation and the group consisted of 40 men and 26 female (39.4%). Age, sex, body measurements and severity of illness were similar between both groups. Primarily SCPC was used as V-V ECMO system and only in 18% (COVID-19-group) and 13% (non-COVID-19-group) of the cases a Cardiohelp-System. All V-V ECMO were set up by percutaneous cannulation and mean V-V ECMO duration was 7.82 (4.41–17.97) days, see Table 1.

**Table1:** Baseline characteristics

	All	Covid19	Non-Covid19	p value
Female	26/66 (39.39%)	4/11 (36.36%)	22/55 (40%)	1.000
28 days survival	40/66 (60.61%)	8/11 (72.73%)	32/55 (58.18%)	0.505
Duration of ICU stay [days]	16.73 (9.36–29.90)	27.89 (11.98–33.23)	14.71 (9.15–24.76)	0.404
Duration of V-V ECMO [days]	7.82 (4.41–17.97)	17.94 (7.8–23.75)	7.49 (4.15–16.34)	0.242
Indication for anticoagulation prior to V-V ECMO	10/66 (15.15%)	1/11 (9.09%)	9/55 (16.36%)	1.000
Height [cm]	174.5 (168.8–180)	175 (170–180)	173 (165–180)	0.430
Body weight [kg]	80 (70.23–90)	85 (79–95)	77.50 (70–90)	0.621
Body-Mass-Index	24.69 (23.51–31.05)	27.78 (25.06–33.87)	24.22 (23.49–30.86)	0.8450
Age [years]	58.36 (46.85–66.23)	59.38 (49.81–61.05)	58.12 (44.35–66.51)	0.808
APACHE II	23.5 (18.75–30)	29 (27–30)	22 (18–31)	0.104
SOFA	12 (10–14.25)	14 (13–16)	12 (9–14)	0.015
RESP Score	1 ((−1)−3)	1 (0–2)	1 ((−2)−4)	0.845

*APACHE II* acute physiology and chronic health score, *RESP Score* respiratory extracorporeal membrane oxygenation survival prediction score, *SOFA* sepsis-related organ failure assessment score

**Fig. 1:**
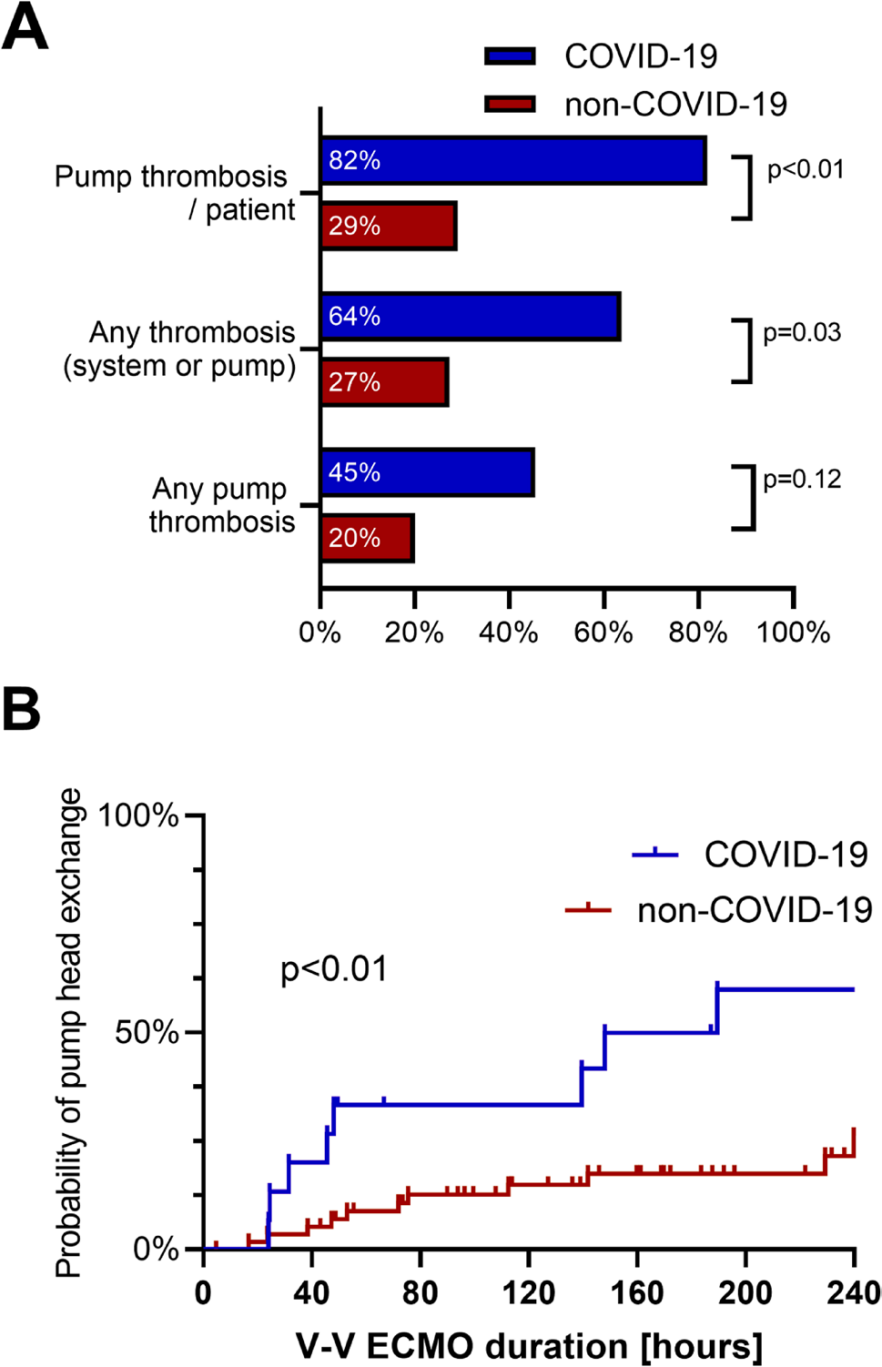
Thrombotic complications in V-V ECMO circuits. **a** Number of exchanges due to thrombus formation in different parts of the extracorporeal circuit. **b** Probability of centrifugal pump exchange due to pump head thrombosis

In total 25 pump head thrombosis requiring exchange in 16 different patients could be registered, nine exchanges for five patients in the COVID-19-group and 16 exchanges for eleven patients in the non-COVID-19 group. Additionally, a total of 13 system exchanges due to thrombus formation were recorded with four in the COVID-19-group and nine in the non-COVID-19 group respectively, see Table 2. The COVID-19-group had a significantly higher number of thrombus formation in the pump head with exchange (*p* < 0.01) and the duration until 50% of patients had a pump head thrombosis was significantly shorter in COVID-19 patients compared to controls (169 h versus 725 h, *p* < 0.01), see Fig. 1.

**Table2:** V-V ECMO related complications and circuit related characteristics as well as laboratory findings at time exchange

	All	Covid19	Non-Covid19	p value
Total V-V ECMO time (days)	916.9	206.1	710.8	
Number of patients with exchange	22 (33.33%)	7 (63.64%)	15 (18.18%)	0.033
Total number of pump changes	25	9	16	
Total number of system changes	13	4	9	
Excluded	10	none	10	
Exchanges/week of therapy	0.29	0.44	0.25	
Flow prior to exchange	2.82 (2.00–3.95)	2.44 (2.01–4.64)	3.00 (1.88–3.68)	0.684
D-Dimers before change	19.42 (9.94–35.2)	35.2 (16.53–35.2)	15.8 (7.52–19.62)	0.005
D-Dimers after change	11.44 (8.57–21.65)	12.78 (9.39–35.2)	10.0 (6.95–16.41)	0.160
Runtime to first change	7.04 (3.39–10.96)	7.9 (5.81–16.77)	5.91 (3–9.55)	0.612
Anticoagulatory target				
Low aPTT target 40–50 s	10 (26.32%)	0 (0%)	10 (40%)	0.008
Medium aPTT target 50–60 s	8 (21.05%)	3 (23.08%)	5 (20%)	1.000
High aPTT target > 60 s	9 (23.68%)	2 (15.38%)	7 (28%)	0.456
Argatroban (aPTT target > 60 s)	11 (28.95%)	8 (61.54%)	3 (12%)	0.003

*V-V ECMO* veno-venous extracorporeal membrane oxygenation, *aPTT* activated partial thromboplastin time

All exchanges occurred during a still sufficiently running V-V ECMO circuit with a median blood flow of 2.9 l/min (3.1 l/min for COVID-19 vs. 2.9 l/min for non-COVID-19). No complete system failure due to fulminant system thrombosis was documented. The system runtime from start of the extracorporeal circulation to the first exchange did not differ between both groups (7.9 (5.81–16.77) days for COVID-19 vs. 5.91 (3–9.55) days for non-COVID-19, *p* = 0.612).

Apart from system and pump head exchanges, additional exchange of two Y-connector changes and two Cytosorb® adsorbers in the COVID-19 group were performed due to thrombosis at the connectors respectively because of a complete thrombosis of the Cytosorb® circuit. In the non-COVID-19 group, no Y-connector or Cytosorb® changes because of thrombotic events were documented. Cytosorb® adsorbers are not routinely used in V-V ECMO patients and indication for adsorber was based on a patient-per-patient level.

**Fig. 2:**
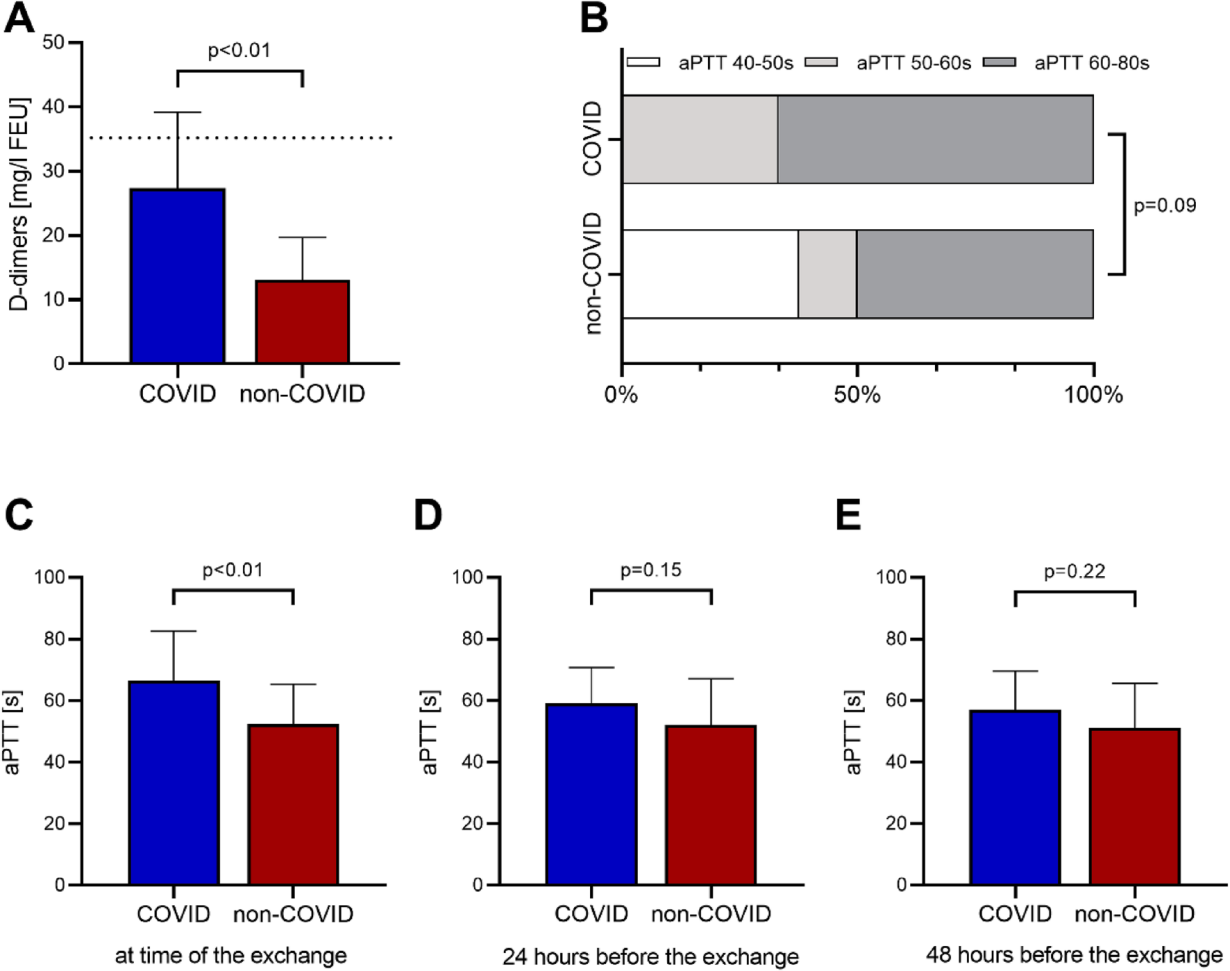
Laboratory findings during the exchange of parts or the whole venovenous extracorporeal membrane oxygenation circuit. **a** D-Dimers levels prior to the exchange, laboratory capped value at 35.2 mg/l. **b** Anticoagulation target prior to the exchange. **c** Last measured activated partial thromboplastin time (aPTT) prior to the exchange. **d** aPTT 24 h prior to the exchange. **e** aPTT 48 h prior to the exchange

When comparing the systemic anticoagulation and the targeted aPTT prior to exchange of the centrifugal pump, targeted aPTT trended to higher target values in the COVID-19 group (*p* = 0.09). The lower target range from 40–50 s was only present in the non-COVID-19 group, see Fig. 2. Also, measured aPTT levels were significantly higher in the COVID-19 group prior to the exchange (62.0 (51.5–83.5) seconds for COVID-19 vs 55.0 (44.5–60.0) seconds for non-COVID-19) whereas measurements 24 and 48 h prior to the exchange showed no significant difference between the groups. D-Dimers were not acquired routinely in the non-COVID-19 group, therefore only 22/25 measurements within 24 h before a change and 18/25 after a change were available. Here, we did see significantly higher D-dimer values in the COVID-19 group prior to change whereas there was no significant difference after change, see Table 2.

## Discussion

In this study, we found a significantly higher rate of thrombotic complications in the ECMO circuit of COVID-19 patients, especially pump head thrombosis, in comparison to non-COVID-19 patients (63.6% vs. 18.2%).

There is only limited data on thrombotic complications in V-V ECMO for both, non-COVID-19 and COVID-19 patients. A retrospective study from 2014 did report an exchange rate due to thrombosis of 27% runs [10]. Other data suggest pump head thrombosis rate of 9% in V-V ECMO [11]. A recent paper on COVID-19 reported three thrombotic occlusions of the centrifugal pump in 2/12 ECMO (25% of runs) patients [9]. A prospective analyse of used circuits in paediatric patients after removal from extracorporeal support did show a higher overall incidence of thrombus formation in the pump head in centrifugal pumps of 41% [12]. Therefore, the incidence of pump head thrombosis detected in our non-COVID-19 cohort is in line with literature. The significant higher number of pump head thrombosis detected in the COVID-19 subgroup is alarming. Thrombus formation in the extracorporeal system, especially the centrifugal pump, if undetected, can result in complete system failure due to complete congestion of the circuit or overheating of the pump shaft.

We noted significantly higher d-dimer values in in COVID-19 patients with thrombus formation in the centrifugal pump compared to control patients. It has been suggested that an incline in d-dimer levels over three days (from 15 to 30 mg/dL) correlates highly with occurrence of thrombosis in ARDS patients on V-V ECMO [13]. Although d-dimers are highly elevated in COVID-19, an increase in d-dimers should not only be attributed to the underlying disease but might also indicate pump head thrombosis.

We found no difference in targeted aPTT in our COVID-19 and non-COVID-19 group with a trend to higher values in the COVID-19 group but aPTT measured at the day of exchange were significantly higher in the COVID-19 group. Interestingly recent data on thrombotic complications in COVID-19 reported high incidence despite systemic anticoagulation [9]. If higher anticoagulation targets can prevent thrombotic events in COVID-19 and are connected to better outcomes in the whole COVID-19 population (and especially in the V-V ECMO Group) has to be addressed in larger trials.

## Limitation

Being a prospective observational, single-center study, inherent limitations and biases are to be presumed and findings are to be considered hypothesis generating. Data on non-COVID-19 patients was collected retrospectively and therefor incomplete documentation is a limiting factor. Also, primarily SCPC systems are used at our center and therefor no rational subgroup analysis for the different systems could be performed.

## Conclusion

Thrombus formation of the extracorporeal circuit in COVID-19 patients is common and potentially life-threatening. Compared to the usual V-V ECMO, the risk is higher and therefor the team should be even more watchful than usual to counteract these kinds of complications.

## Abbreviations

aPTT: Activated partial thromboplastin time
ARDS: Acute respiratory distress syndrome
COVID-19: Coronavirus disease 2019
ICU: Intensive care unit
SARS-CoV-2: Severe acute respiratory syndrome coronavirus 2
V-V ECMO: Venovenous extracorporeal membrane oxygenation
